# Neurogranin as a Synaptic Biomarker in Mild Traumatic Brain Injury: A Systematic Review of Diagnostic and Pathophysiological Evidence

**DOI:** 10.3390/proteomes13030046

**Published:** 2025-09-19

**Authors:** Ioannis Mavroudis, Foivos Petridis, Eleni Karantali, Dimitrios Kazis

**Affiliations:** 1 Department of Neuroscience, Leeds Teaching Hospitals, NHS Trust, Leeds LS1 3EX, UK; 2 Department of Neurosciences, University of Leeds, Leeds LS2 9JT, UK; 3 Third Department of Neurology, Aristotle University of Thessaloniki, 57010 Thessaloniki, Greece; f_petridis83@yahoo.gr (F.P.); dimitrios.kazis@gmail.com (D.K.); 4 Department of Neurology, University of Alexandroupolis, 69100 Alexandroupoli, Greece; lena.kar@outlook.com

**Keywords:** neurogranin, mild traumatic brain injury, biomarker, synaptic protein, exosomes, concussion

## Abstract

Neurogranin (NRGN), a synaptic protein essential for plasticity and memory function, is gaining recognition as a promising biomarker for mild traumatic brain injury (mTBI). This systematic review brings together findings from six studies that measured neurogranin levels in biofluids—including serum, cerebrospinal fluid (CSF), plasma, and exosomes—during both the acute and chronic phases following injury. In the acute phase of mTBI, elevated levels of neurogranin were consistently observed in serum samples, suggesting its potential as a diagnostic marker. These increases appear to reflect immediate synaptic disturbances caused by injury. In contrast, studies focusing on the chronic phase reported a decrease in exosomal neurogranin levels, pointing to ongoing synaptic dysfunction well after the initial trauma. This temporal shift in neurogranin expression highlights its dual utility—both as an early indicator of injury and as a longer-term marker of synaptic integrity. However, interpreting these findings is not straightforward. The studies varied considerably in terms of sample type, timing of measurements, and control for potential confounding factors such as physical activity. Such variability makes direct comparisons difficult and may influence the outcomes observed. Additionally, none of the studies included proteoform-specific analyses of neurogranin, an omission that limits our understanding of the molecular changes underlying mTBI-related synaptic alterations. Due to heterogeneity across study designs and outcome measures, a meta-analysis could not be performed. Instead, a narrative synthesis was conducted, revealing consistent patterns in neurogranin dynamics over time and underscoring the influence of biofluid selection on measured outcomes. Overall, the current evidence supports neurogranin’s potential as both a diagnostic and mechanistic biomarker for mTBI. Yet, to fully realize its clinical utility, future research must prioritize standardized protocols, the inclusion of proteoform profiling, and rigorous longitudinal validation studies.

## 1. Introduction

Mild traumatic brain injury (mTBI), commonly referred to as concussion, is a prevalent neurological condition that affects millions globally, particularly among military personnel, athletes, and individuals involved in accidents or falls [1]. While most mTBI cases resolve within weeks, a substantial subset of patients experience persistent symptoms such as headaches, cognitive impairment, mood disturbances, and sleep disruptions, a condition collectively termed post-concussion syndrome (PCS) [2]. The challenge of diagnosing and monitoring mTBI largely stems from the absence of objective biomarkers that reliably reflect underlying neuropathological changes, particularly in patients with normal neuroimaging results [3].

Recent advances in neurobiology have shifted focus toward synaptic dysfunction as a central mechanism in both acute and chronic stages of mTBI. Synaptic proteins, given their critical role in cognitive processes, are emerging as promising biomarkers for TBI-related neuronal stress and injury. One such protein is neurogranin (NRGN), a postsynaptic calmodulin-binding protein predominantly expressed in dendritic spines within the cortex and hippocampus [4]. Neurogranin plays a pivotal role in synaptic plasticity and long-term potentiation, processes essential for memory and learning [5].

While neurogranin has been extensively studied as a cerebrospinal fluid (CSF) biomarker in Alzheimer’s disease and other neurodegenerative conditions [6,7], its utility in the context of mTBI is a relatively nascent field. The rationale for investigating neurogranin in mTBI is twofold. First, mTBI often results in synaptic disruption without gross neuronal loss, and therefore, markers like neurogranin that reflect subtle synaptic changes could be highly sensitive [8]. Second, neurogranin has the potential to differentiate between transient post-injury responses and chronic synaptic dysfunction associated with persistent symptoms or neurodegeneration.

Four key studies illustrate the current landscape of neurogranin research in mTBI. Shahim et al. examined cerebrospinal fluid biomarkers in professional ice hockey players with chronic PCS and found that while neurofilament light protein and amyloid-β were significantly altered, neurogranin was included in the biomarker panel but did not show strong associations [9]. This study suggests that while synaptic pathology is implicated in PCS, neurogranin levels in CSF may not reliably reflect chronic changes or may require longitudinal profiling to establish trends.

In contrast, Di Battista et al. demonstrated that intense physical exertion itself can acutely elevate neurogranin levels in plasma [10]. This study, involving healthy recreational athletes undergoing high-intensity interval training (HIIT), revealed transient increases in neurogranin and other brain injury-associated biomarkers, highlighting exercise as a major confounding variable in biomarker interpretation. Importantly, neurogranin levels showed an attenuated response following two weeks of training, suggesting adaptive neuroplastic changes. These findings underscore the necessity of accounting for recent physical activity when evaluating neurogranin as a biomarker for mTBI.

A more clinically focused study by Çevik et al. investigated serum levels of neurogranin, GFAP, and S100B in 48 mTBI patients stratified by the presence or absence of CT-detectable lesions [11]. Neurogranin levels were significantly elevated in CT-positive patients compared to CT-negative controls, indicating its potential role as an acute biomarker for structural brain injury. With a reported sensitivity of 83.3% and specificity of 58.3% at a cut-off of 1.87 ng/mL, neurogranin demonstrated comparable or superior diagnostic performance relative to traditional biomarkers. This study provides preliminary evidence for neurogranin’s diagnostic relevance in emergency settings and its utility in risk stratification.

Adding a novel dimension, Winston et al. analyzed neuron-derived and astrocyte-derived exosomes from plasma samples of military service members with chronic mTBI [12]. Using immunoaffinity-based isolation techniques, the authors found that neurogranin levels in both neuronal and astrocytic exosomes were significantly reduced in individuals with mTBI compared to healthy controls. Furthermore, exosomal cargo from mTBI participants was cytotoxic to neuron-like cells in vitro, suggesting that neurogranin depletion may reflect pathogenic mechanisms beyond mere synaptic marker loss. This study highlights the value of exosomal biomarkers in detecting persistent synaptic alterations and their potential to inform mechanistic insights into long-term consequences of mTBI.

Taken together, these findings suggest a complex temporal and matrix-dependent behavior of neurogranin in mTBI. Elevated levels in acute serum samples may reflect synaptic shedding or early excitotoxic responses, while decreased levels in chronic exosomes may indicate ongoing synaptic degradation or loss of plasticity. The presence of exercise-induced fluctuations further complicates interpretation and necessitates standardized protocols for sample timing and activity control.

Given the heterogeneity of study designs, sample types (serum, plasma, CSF, exosomes), and timepoints post-injury, a meta-analysis is currently not feasible. However, a systematic review offers a valuable opportunity to synthesize existing evidence, identify knowledge gaps, and propose standardized methodologies for future research.

This systematic review addresses the following research question: Is neurogranin a reliable biomarker for diagnosis, prognosis, and mechanistic understanding of mild traumatic brain injury across different biofluids and timepoints? Our aim is to (1) summarize the evidence on neurogranin as a biomarker in mild traumatic brain injury across different biological matrices, (2) assess its diagnostic and prognostic potential in acute and chronic phases, (3) evaluate confounding variables such as physical activity, and (4) propose a roadmap for future validation and clinical translation.

## 2. Materials and Methods

This systematic review was conducted following the PRISMA (Preferred Reporting Items for Systematic Reviews and Meta-Analyses) guidelines. The review protocol has been registered with Prospero with reference: 1068726.

### Eligibility Criteria

We included original research studies that met the following criteria:

- Population: Human participants diagnosed with mild traumatic brain injury (GCS 13–15).
- Biomarker: Neurogranin (NRGN) measured in blood, CSF, or exosomes.
- Outcome: Diagnostic, prognostic, or mechanistic insights into mTBI.
- Study Design: Cohort, case–control, cross-sectional, or interventional studies.
- Language: English only.

We excluded:

- Animal studies;
- Studies not reporting neurogranin measurements;
- Reviews, editorials, or case reports.

Information Sources and Search Strategy Searches were conducted in PubMed, Scopus, Web of Science, and Google Scholar for articles published up to May 2025. Search terms included: (“neurogranin” OR “NRGN”) AND (“mild traumatic brain injury” OR “mTBI” OR “concussion”) AND (“biomarker” OR “blood” OR “serum” OR “CSF” OR “exosome”).

Study Selection and Data Extraction Two reviewers independently screened titles and abstracts for eligibility. Full texts were retrieved for shortlisted articles and assessed for inclusion. Discrepancies were resolved through consensus. Data extracted included: author/year, population/sample size, sample type, time post-injury, neurogranin measurement method, outcomes, and key findings.

Quality Assessment Study quality and risk of bias were assessed using the Newcastle–Ottawa Scale (NOS) for observational studies. Factors considered included selection criteria, comparability of cohorts, and outcome assessment.

Synthesis Given the heterogeneity in methodologies, populations, and biomarkers, a narrative synthesis approach was used to summarize trends and interpret findings across studies.

## 3. Results

**Figure 1:**
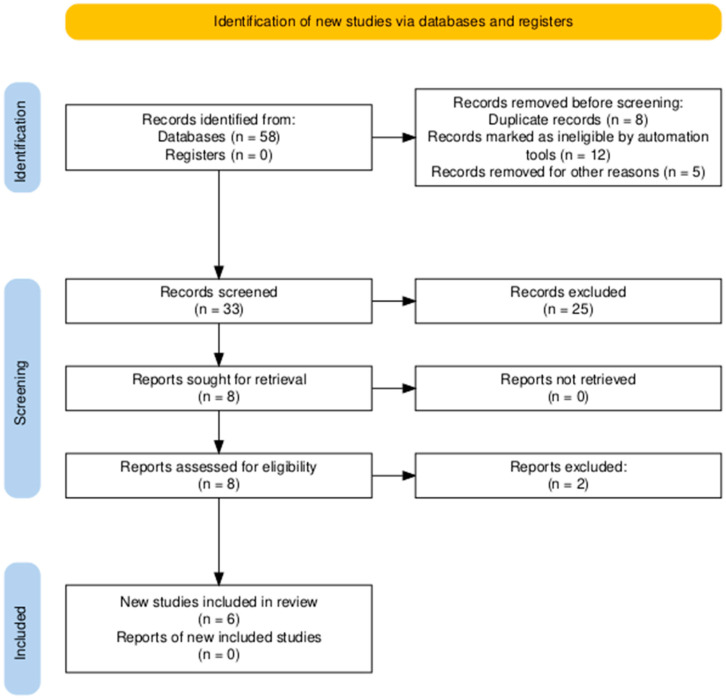
PRISMA flow diagram showing the study selection process. A total of 58 records were identified, with 6 studies ultimately included in the systematic review.

**Figure 2:**
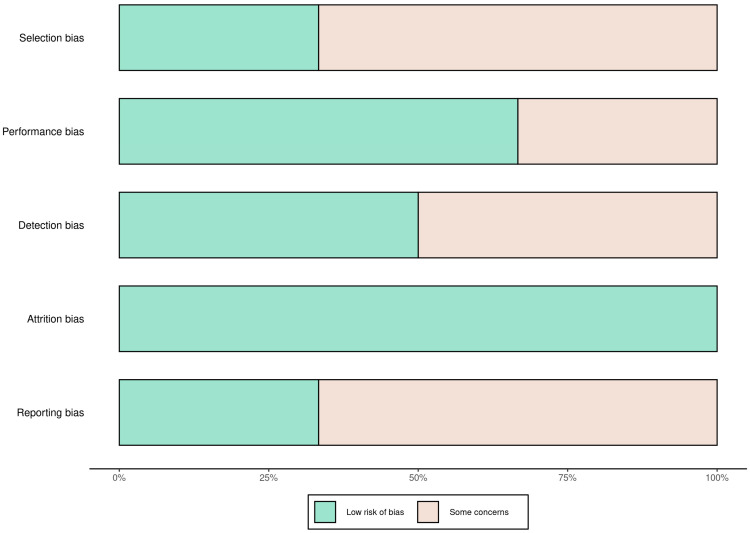
Risk of bias summary across studies using five domains. Most studies had low risk of attrition and performance bias, with moderate concerns in selection, detection, and reporting domains.

**Figure 3:**
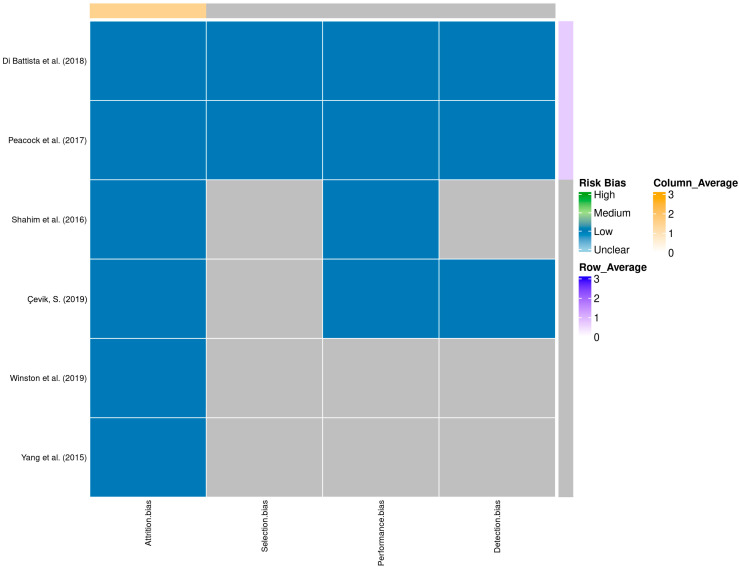
Heatmap of domain-specific risk of bias assessments across studies [9,10,11,12,13,14]. Shades represent different levels of risk, with row and column averages displayed to visualize aggregate quality scores.

A total of 58 records were identified through database searching. Following the removal of duplicates and automated exclusions, 33 records were screened, of which 8 full-text articles were reviewed. Six studies met the eligibility criteria and were included in this systematic review (Figure 1, Table 1). A risk of bias assessment was performed across five methodological domains. Most studies showed low risk in performance and attrition domains, while moderate concerns were noted in selection, detection, and reporting domains (Figure 2 and Figure 3).

Among the studies included, Shahim et al. conducted a multicenter investigation evaluating cerebrospinal fluid biomarkers in professional ice hockey players who had experienced persistent post-concussive symptoms for more than three months [9]. Neurogranin was one of several biomarkers assessed in this chronic PCS population. However, the study did not find neurogranin to be significantly altered between players with PCS and healthy controls. Other biomarkers, particularly neurofilament light and amyloid-β, exhibited more consistent associations with chronic symptoms. The absence of a significant change in neurogranin in this setting may suggest limited sensitivity of CSF neurogranin in chronic phases of mTBI or that its dynamics require more nuanced, perhaps longitudinal, assessment to capture relevant changes.

In a contrasting line of inquiry, Di Battista et al. [10] explored the influence of high-intensity interval training (HIIT) on neurogranin and other biomarkers in plasma samples of healthy, recreationally active individuals. Their findings indicated that neurogranin levels were significantly elevated immediately after a single bout of HIIT, demonstrating the biomarker’s sensitivity to acute physical exertion. Interestingly, following a six-session training protocol over two weeks, the acute elevations in neurogranin were attenuated, suggesting an adaptive or habituated response of the synaptic network to sustained exercise stress. This study underscores the need to control for recent physical activity when interpreting neurogranin levels, as elevations might not solely reflect injury but rather reflect transient physiological stress.

Çevik et al. [11] provided evidence from a cross-sectional study involving emergency department patients with mild TBI. These patients were categorized based on computed tomography findings into CT-positive and CT-negative groups. Neurogranin levels measured in serum were significantly higher in CT-positive patients, with values demonstrating a strong statistical distinction compared to CT-negative individuals. The researchers calculated a diagnostic sensitivity of 83.3% at a cut-off of 1.87 ng/mL, though specificity was more modest. This suggests that neurogranin could serve as a useful adjunctive biomarker in early stratification of patients at higher risk of intracranial pathology, potentially guiding decisions on imaging or closer observation.

Winston et al. [12] investigated neurogranin in a more mechanistic context by isolating neuron-derived and astrocyte-derived exosomes from plasma of military service members with a history of mTBI. Notably, neurogranin levels in both exosomal populations were found to be significantly lower in individuals with chronic mTBI compared to controls. Moreover, neurogranin-depleted exosomes from affected individuals exhibited cytotoxic effects on neuron-like cell cultures, suggesting a direct pathophysiological role for altered exosome cargo in sustaining or exacerbating neuronal damage. These findings position neurogranin not only as a biomarker of synaptic health but also as a possible actor in the pathology of chronic brain injury.

When considered collectively, these studies illustrate that neurogranin’s behavior as a biomarker in mTBI is multifaceted and highly context-dependent. In the acute setting, especially in serum samples, neurogranin tends to be elevated, likely reflecting acute synaptic perturbation or excitotoxic release. Conversely, in the chronic phase, particularly in exosomal compartments, reduced neurogranin levels may signal enduring synaptic dysfunction or structural degradation. The role of confounding variables such as recent exercise is also prominent and must be rigorously controlled in future studies. While the heterogeneity of current data limits meta-analytical synthesis, the emerging narrative underscores neurogranin’s dual potential as both a diagnostic and mechanistic indicator in the evolving understanding of mTBI pathology.

In addition, Peacock et al. (2017) presented compelling evidence from the HeadSMART study, a large-scale, multicenter observational study that evaluated 662 patients presenting with head injury [13]. This study investigated a panel comprising neurogranin (NRGN), neuron-specific enolase (NSE), and metallothionein 3 (MT3) in serum samples taken within 24 h post-injury. NRGN levels were found to be elevated in a time-dependent manner, particularly between 2 and 6 h post-injury, with trends suggesting continued elevation up to 24 h. When analyzed using a logistic regression and random forest model that included age and sex as covariates, the three-biomarker panel demonstrated a high diagnostic accuracy (AUC 0.91), with sensitivity reaching 98% and specificity 72%. These findings underscore the clinical utility of neurogranin, particularly when interpreted in the context of multiparametric models. The HeadSMART study highlights the value of incorporating neurogranin into diagnostic algorithms for early detection of mTBI, especially in settings where imaging findings may be equivocal or absent.

Yang et al. (2015) [14] further substantiated the utility of neurogranin as a circulating biomarker by developing a novel and highly sensitive ELISA platform. This assay was employed to evaluate serum samples from 76 TBI patients and 150 controls. The study reported significantly elevated neurogranin levels in the TBI cohort (median 0.18 ng/mL) compared to controls (median 0.02 ng/mL), with a robust statistical difference (*p* < 0.0001) and an AUC of 0.72. While neurogranin concentrations did not significantly distinguish patients with intracranial hemorrhage from those without, this result reflects its sensitivity as a general indicator of brain injury rather than as a marker of lesion severity. These findings align with the hypothesis that neurogranin, as a small synaptic protein, crosses the blood–brain barrier efficiently during early neurotrauma and offers significant diagnostic potential in the emergency department setting.

## 4. Discussion

The relevance of neurogranin in mTBI also resonates with findings from Alzheimer’s disease and related disorders. Multiple studies have demonstrated that neurogranin measured in CSF and blood exosomes reflects synaptic dysfunction and correlates with cognitive decline [15,16]. Our own meta-analysis confirmed elevated CSF neurogranin in Alzheimer’s disease and mild cognitive impairment, although other work suggests that neurogranin may not be entirely disease-specific [17,18]. Taken together, these findings reinforce neurogranin’s broader role as a marker of synaptic pathology and highlight its potential to link acute synaptic injury after mTBI with long-term risk of neurodegenerative disease. This systematic review of neurogranin (NRGN) as a biomarker in mild traumatic brain injury (mTBI) reveals a promising but complex picture. Across diverse populations, methodologies, and timepoints post-injury, neurogranin consistently demonstrates sensitivity to synaptic alterations, underscoring its potential value in both acute and chronic stages of mTBI. Unlike traditional biomarkers such as GFAP and NFL, which reflect glial and axonal injury, respectively, neurogranin highlights the synaptic dimension of brain trauma, which is highly relevant for cognitive outcomes and risk of later neurodegeneration. However, the variation in biological matrices, timing of sampling, and external influences such as physical activity introduces interpretive challenges that must be carefully considered in future clinical applications.

The most robust evidence supporting neurogranin’s clinical utility comes from studies evaluating its presence in acute blood samples following mTBI. Both Çevik et al. and Yang et al. [11,12] reported significant elevations in serum neurogranin levels shortly after injury, distinguishing mTBI patients from controls with good sensitivity and moderate specificity. Similarly, the HeadSMART study by Peacock et al. [13] validated neurogranin as part of a multi-marker panel (NRGN, NSE, MT3), achieving excellent diagnostic performance (AUC 0.91) and highlighting its potential as a component of early emergency department triage algorithms. These findings emphasize neurogranin’s acute responsiveness to brain injury and support its inclusion in point-of-care diagnostic strategies, especially in environments where imaging resources are limited or inconclusive. Taken together, these acute studies suggest that neurogranin may serve as a rapid, accessible biomarker, particularly when combined with complementary markers to improve specificity.

On the other hand, chronic stages of mTBI appear to reveal a different neurogranin signature. The study by Winston et al. [12], which assessed neurogranin in neuron- and astrocyte-derived exosomes, found significantly reduced levels in individuals with persistent post-concussive symptoms months after injury. This depletion was associated with cytotoxic effects in neuronal cell cultures, suggesting that neurogranin may play an active role in long-term synaptic dysregulation. Similarly, the study by Shahim et al. [9], although less conclusive, indicated that chronic changes in neurogranin may not always be detectable in cerebrospinal fluid, underscoring the importance of matrix-specific dynamics and suggesting that exosomal profiling may be more sensitive than CSF in chronic phases of mTBI.

A critical consideration raised by Di Battista et al. [10] is the influence of physical activity on neurogranin levels. Their findings demonstrate that high-intensity exercise alone can elevate plasma neurogranin, with adaptive attenuation over time. This physiological response complicates the interpretation of elevated neurogranin in the context of mTBI, particularly in athletic or military populations. Future biomarker studies should therefore incorporate standardized protocols that control for exercise and exertion prior to sampling, as this represents a major potential confounder.

An important, yet unaddressed, dimension across current studies is the role of neurogranin proteoforms. Neurogranin exists in multiple forms, including phosphorylated and truncated variants, which may have distinct functional and pathological implications. However, none of the reviewed studies assessed proteoform-specific expression or post-translational modifications, relying instead on total neurogranin quantification via ELISA or immunoassays. This may obscure critical information about specific mechanisms of synaptic dysfunction. For example, phosphorylated neurogranin has been linked to altered calmodulin signaling and impaired synaptic plasticity. The absence of proteoform-specific data is a major limitation, and future studies should employ proteomic technologies such as mass spectrometry to characterize the molecular heterogeneity of neurogranin in mTBI. A further methodological concern relates to the analytical platforms used for neurogranin quantification. Most of the available studies employed ELISA or related immunoassays that rely on antibodies targeting the C-terminal fragment of neurogranin. While widely accessible, this approach introduces important limitations: truncated or phosphorylated variants of the protein may not be recognized, resulting in underestimation or inaccurate quantitation of total neurogranin burden. In particular, the omission of truncated neurogranin species is problematic, as these fragments may represent functionally distinct states of synaptic pathology. The restricted epitope recognition of current assays therefore constrains both diagnostic accuracy and mechanistic interpretation. To improve reliability, future studies should develop or adopt antibodies that target multiple epitopes or combine immunoassays with proteoform-sensitive technologies.

In this context, liquid chromatography coupled with tandem mass spectrometry (LC-MS/MS) has emerged as the gold standard for protein quantification in biological matrices. This method not only offers superior sensitivity and specificity compared to conventional immunoassays but also allows for the simultaneous detection of full-length neurogranin and its proteoforms, including truncated or post-translationally modified variants. LC-MS/MS has already been successfully applied in Alzheimer’s disease and related neurodegenerative disorders to quantify neurogranin in cerebrospinal fluid and plasma. Extending this methodology to mTBI cohorts would therefore be a logical next step, providing more accurate abundance measures, reducing assay-related bias, and enabling mechanistic insights through proteoform-level resolution.

Several limitations constrain the generalizability of current findings. Sample sizes in most studies were modest, and study designs varied widely in terms of biomarker collection timing, injury characterization, and analytic platforms. Control for potential confounders, such as physical activity, comorbid conditions, and medication use, was inconsistent. The absence of longitudinal sampling in most cohorts limits understanding of neurogranin’s temporal dynamics, and few studies explored how its levels correlate with clinical recovery, symptom persistence, or neurocognitive outcomes. Additionally, inter-study variability in matrix type (serum, CSF, plasma, or exosomes) further complicates direct comparison or meta-analytic synthesis. Publication bias cannot be excluded, as several datasets originated from specialized biomarker research groups, potentially skewing available evidence.

Most importantly, the current evidence positions neurogranin within a broader pathophysiological context: mTBI is increasingly recognized as a risk factor for dementia and neurodegenerative diseases. Neurogranin has already been established as a sensitive marker of synaptic degeneration in Alzheimer’s disease, and its altered dynamics after mTBI may represent a mechanistic link between acute synaptic injury and later-life cognitive decline. This observation strengthens the rationale for including neurogranin in longitudinal studies that track both short-term recovery and long-term risk of neurodegeneration.

Beyond traumatic brain injury, neurogranin has been repeatedly validated as a marker of synaptic dysfunction in several neurodegenerative disorders. In Alzheimer’s disease and mild cognitive impairment, elevated cerebrospinal fluid neurogranin levels correlate strongly with cognitive decline and progression, while blood-derived exosomal neurogranin has shown promise as a minimally invasive biomarker of early synaptic degeneration. Increased neurogranin concentrations have also been reported in frontotemporal dementia and Parkinson’s disease with dementia, supporting the view that it reflects a generalizable mechanism of synaptic injury rather than a disease-specific signal. By situating neurogranin within this broader neurodegenerative framework, mTBI can be conceptualized not only as an acute synaptic insult but also as a potential initiating event that converges with later-life neurodegenerative pathways. This expanded scope highlights neurogranin’s potential as a cross-disease biomarker of synaptic integrity and strengthens its translational relevance.

Taken together, the results of this review suggest a dual role for neurogranin. In the acute setting, elevated serum levels reflect immediate synaptic disturbance and can aid in diagnosis. In the chronic context, particularly in exosomal compartments, reduced neurogranin may signal prolonged synaptic degeneration or failure of neuronal recovery mechanisms. This biphasic behavior parallels the evolving pathophysiology of mTBI, transitioning from acute excitotoxicity to longer-term circuit reorganization or degeneration. It also underscores neurogranin’s value as both a state and trait marker—responsive to current injury and informative of longer-term prognosis.

To move the field forward, future research should prioritize (1) the development of standardized sampling protocols that control for activity and injury timing, (2) inclusion of proteoform-specific analysis of neurogranin to improve mechanistic insights, and (3) longitudinal studies in large, diverse populations to assess neurogranin’s utility in tracking recovery or predicting poor outcomes. Moreover, integration of neurogranin with established biomarkers such as GFAP and NFL into multi-analyte panels, potentially supported by machine learning approaches, may enhance diagnostic accuracy and predictive power.

This systematic review has several limitations that must be acknowledged. First, the heterogeneity across included studies in terms of neurogranin sampling matrices (serum, CSF, plasma, exosomes), timepoints post-injury, and assay techniques precluded meta-analysis and limited the ability to perform direct comparisons or pooled effect estimates. Second, most studies were small in sample size and involved single-center cohorts, which may affect generalizability. Third, methodological variability—such as inconsistent control for confounding factors like physical activity, medication use, or comorbidities—could have influenced neurogranin measurements and their interpretation. Furthermore, none of the studies evaluated neurogranin proteoforms, such as phosphorylated or cleaved variants, which may offer additional mechanistic and diagnostic insights. The lack of longitudinal data in most reports also prevented assessment of neurogranin’s temporal dynamics in recovery or chronic symptomatology. Additionally, publication bias cannot be excluded, particularly since several datasets originated from research groups known to study brain injury biomarkers, potentially skewing findings toward positive results. Finally, the absence of formal risk of bias assessments across all domains in some studies and limited external validation of findings suggest that further high-quality research is needed before neurogranin can be adopted in clinical practice.

## Figures and Tables

**Table 1:** This table summarizes four studies evaluating neurogranin (NRGN) as a biomarker in mild traumatic brain injury (mTBI), detailing sample types, populations, post-injury timing, key findings, and interpretations. It highlights the variable behavior of NRGN across biological matrices and timepoints.

Study	Sample Type	Population	Time Post-Injury	Sample Size	Key Findings	Strengths/Limitations
Shahim et al. (2016) [9]	Cerebrospinal fluid (CSF)	Professional athletes with persistent PCS vs. controls	>3 months	*n* = 28 PCS, *n* = 19 controls	Neurogranin not significantly altered; NFL and amyloid-β more robust biomarkers	High-quality CSF biomarker panel; small sample; limited sensitivity of CSF neurogranin
Di Battista et al. (2018) [10]	Plasma	Healthy athletes undergoing high-intensity interval training (not TBI)	Acute, post-exercise	*n* = 16	Acute increases in neurogranin post-exercise; attenuated after training	Identified exercise as confounder; not TBI-specific
Çevik et al. (2019) [11]	Serum	Emergency department patients with mTBI, CT-positive vs. CT-negative	<4 h	*n* = 48	Neurogranin significantly higher in CT+ patients (5.79 vs. 2.95 ng/mL, *p* = 0.001); sensitivity 83%, specificity 58%	First acute diagnostic study; modest sample; moderate specificity
Winston et al. (2019) [12]	Plasma-derived neuronal and astrocytic exosomes	Military personnel with chronic mTBI vs. controls	~5 months (mean ~151 days)	*n* = 32 mTBI, *n* = 32 controls	Reduced exosomal neurogranin; exosomes from mTBI patients cytotoxic to neuronal cultures	Novel exosome-based approach; cross-sectional design; small sample size
Peacock et al. (2017)—HeadSMART study [13]	Serum	Multicenter emergency department head injury cohort	<24 h	*n* = 662	Neurogranin (with NSE and MT3) showed high diagnostic accuracy (AUC 0.91; sensitivity 98%, specificity 72%)	Large multicenter study; biomarker panel approach; not neurogranin-specific
Yang et al. (2015) [14]	Serum	TBI patients vs. controls	Acute	*n* = 76 TBI, *n* = 150 controls	Neurogranin significantly higher in TBI (median 0.18 vs. 0.02 ng/mL, *p* < 0.0001); AUC 0.72	Novel ELISA platform; included some moderate TBI; no imaging stratification

## Data Availability

No new data were generated or analyzed in this study. All data discussed are derived from previously published studies, which are cited and available in the public domain.
